# Modeling Transcranial Direct-Current Stimulation-Induced Electric Fields in Children and Adults

**DOI:** 10.3389/fnhum.2018.00268

**Published:** 2018-07-03

**Authors:** Patrick Ciechanski, Helen L. Carlson, Sabrina S. Yu, Adam Kirton

**Affiliations:** ^1^Calgary Pediatric Stroke Program, University of Calgary, Calgary, AB, Canada; ^2^Department of Neurosciences, University of Calgary, Calgary, AB, Canada; ^3^Departments of Pediatrics and Clinical Neurosciences, University of Calgary, Calgary, AB, Canada

**Keywords:** current modeling, tDCS, motor, pediatrics, FEM, children

## Abstract

Transcranial direct-current stimulation (tDCS) is a form of non-invasive brain stimulation that induces electric fields in neuronal tissue, modulating cortical excitability. Therapeutic applications of tDCS are rapidly expanding, and are being investigated in pediatrics for various clinical conditions. Anatomical variations are among a host of factors that influence the effects of tDCS, and pronounced anatomical differences between children and adults suggest that induced electric fields may be substantially different across development. The aim of this study was to determine the strength and distribution of tDCS-induced electric fields across development. Typically developing children, adolescents, and adults were recruited. Individualized finite-element method modeling of primary motor cortex (M1) targeting tDCS was performed. In the largest pediatric sample to date, we found significantly higher peak and mean M1 electric field strength, and more expansive electric field spread for children compared to adults. Electric fields were often comparable between adolescents and adults. Our results suggest that these differences may be associated with age-related differences in skull and extra-axial space thickness, as well as developmental changes occurring in gray and white matter. Individualized current modeling may be a valuable tool for personalizing effective doses of tDCS in future pediatric clinical trials.

## Introduction

Transcranial direct-current stimulation (tDCS) is a form of non-invasive brain stimulation commonly investigated as a neuromodulator in healthy and clinical populations. Through the induction of electric fields, weak direct-current passing through the brain alters cortical excitability. Behavioral changes are evident when modulating cortical excitability. For example, tDCS targeting the motor cortex may enhance motor skill acquisition in healthy subjects (Buch et al., 2017) and facilitate motor rehabilitation in subjects with post-stroke hemiparesis (Hummel et al., 2005; Kirton et al., 2016). A wide and rapidly expanding range of clinical and neurophysiological applications are described (Lefaucheur, 2016). As an emerging therapeutic tool, tDCS is also increasingly applied in the developing brain (Hameed et al., 2017) even though the mechanistic investigations performed in adults over the past 15 years are virtually absent in pediatric populations.

Early neurophysiological studies suggest that the effects of tDCS may be different in children as compared to adults (Moliadze et al., 2015, 2018). Clinical studies of motor effects of tDCS in children suggest that tDCS may enhance motor skill acquisition, however, the mechanisms underlying this enhancement may differ from adults (Reis et al., 2009; Ciechanski and Kirton, 2016a), also suggesting unique effects. For example, in adults, tDCS enhances motor learning primarily by altering offline consolidation processes (Reis et al., 2009), whereas in children, modulation of online practice effects is the primary mode by which tDCS enhances hand function (Ciechanski and Kirton, 2016a). The young developing brain undergoes substantial and rapid changes in excitatory and inhibitory networks, both of which tDCS is thought to influence (Hameed et al., 2017). Therefore, direct translation of mechanistic studies from adult to pediatrics may not be entirely valid. Despite a relative paucity of evidence, tDCS is increasingly applied across clinical populations, most notably in cerebral palsy where treatments are currently limited and evidence of enhanced motor learning makes tDCS a logical possibility. Some tDCS clinical trials are controlled, blinded, randomized trials with safety outcomes (Kirton et al., 2017) but many others are not (Ciechanski and Kirton, 2016b). Other clinical uses include seizure control, autism-spectrum disorder, and attention-deficit hyperactivity disorder (Lefaucheur, 2016). With such pediatric applications expanding rapidly, there is a pressing need to better define how tDCS effects may differ in the developing brain.

Advances in computational finite-element method (FEM) modeling allow for predictions of electric field strength and distribution induced by tDCS (Datta et al., 2013). For example, cross-sectional investigations have quantified tDCS-induced electric fields in groups of healthy adults, revealing cortical regions that experience consistent or variable electric field strength (Laakso et al., 2016). These sources of variability include idiosyncrasies of gyral/sulcal geometry, white (WM) and gray matter (GM) architecture, variations in skull thickness and shunting of current through highly conductive cerebrospinal fluid (CSF), among many other factors that complicate current modeling (Datta et al., 2009; Opitz et al., 2015). These anatomical factors display the greatest variability in children and adolescents, as these populations are actively developing, compared to the relatively stable anatomy of young to middle-aged adults.

Small current modeling case series suggest that tDCS-induced electric fields may be stronger in children compared to adults (Minhas et al., 2012). These varying effects are postulated to relate to multiple anatomical differences associated with younger age. Multiple small case series have suggested similar findings in pediatric tDCS application (Kessler et al., 2013; Gillick et al., 2014; Parazzini et al., 2014, 2015; Fiocchi et al., 2016). These case reports are not sufficiently powered to capture the full extent of potential electric field variability in pediatrics, owing to ongoing changes in brain neuroanatomy and morphology that occur throughout development. It is imperative that electric fields be modeled cross-sectionally, as subject-specific modeling is imperative to maximizing the safety profile and therapeutic potential of tDCS (Dmochowski et al., 2013). Advancing such understanding may facilitate tDCS study design across broader applications, while promoting greater precision and personalized approaches to non-invasive neuromodulation.

This project aimed to investigate the effects of primary motor cortex (M1) targeting tDCS montages on the strength and distribution of induced electric fields in pediatric populations. Using parameters defined by typical M1 targeting tDCS montages, we modeled electric fields in typically developing children, adolescents, and adults. We hypothesized that children would experience stronger and more expansive electric fields compared to adults. Based on previous reports examining factors that influenced electric fields induced by tDCS (Opitz et al., 2015), we predicted that skull and CSF thickness would largely influence the strength of electric fields, based on their ability to block and shunt electric current, respectively.

## Materials and Methods

### Participants

Typically developing children were recruited through an established healthy controls recruitment program at the Alberta Children’s Hospital. Healthy adult volunteers (aged > 20 years) were recruited via word of mouth. All participants were right handed, had no MRI contraindications, and denied any neurodevelopmental or neuropsychiatric conditions. The younger participant group was further subdivided into two groups based on age; children (aged 6.0–12.9 years) and adolescents (aged 13.0–19.0 years) using a threshold of 13 years. This threshold was chosen since it approximated the central tendency of age for the sample < 20 years of age (mean = 12.9, median = 12.5). The upper threshold of age for the adolescent group (age > 20 years) was chosen since plateaus are thought to have typically been reached in development by this age (Lebel et al., 2008; Brain Development Cooperative Group, 2012) though we do acknowledge that developmental changes do occur after this period. Written parental informed consent and participant assent was obtained for pediatric participants. Informed consent was obtained from adult participants. This study was approved by the Conjoint Health Research Ethics Board, University of Calgary.

### Imaging

Images were acquired in a single scanning session at the ACH Diagnostic Imaging Suite using a 3.0 Tesla GE Discovery MR750w MRI scanner (GE Healthcare; Waukesha, WI, United States) with an MR Instruments (Minnetonka, MN, United States) 32-channel receive-only head coil. High-resolution anatomical T1-weighted fast spoiled gradient echo (FSPGR) images were acquired in the axial plane [166 slices, no skip; voxel size = 1.0 mm isotropic; repetition time (TR) = 8.5 ms; echo time (TE) = 3.2 ms; flip angle = 11°; field of view (FOV) = 256; matrix = 256 × 256]. T2-weighted images were acquired in the axial plane [36 slices, no skip; voxel size = 0.45 mm × 0.45 mm; slice thickness = 3.6 mm; TR/TE = 6187/80 ms; FOV = 230 mm; matrix = 512 × 512]. No head motion correction was performed for the aforementioned anatomical scans. For a subset of participants (adults *N* = 13, adolescents *N* = 15, children *N* = 17), diffusion weighted images (DWIs) were acquired in 32 non-collinear directions (*b* = 750 or 900 s/mm^2^, four volumes using *b* = 0 s/mm^2^, voxels = 2.2–2.5 mm^3^ isotropic, duration = 6 min, TR/TE = 11.5 s/70 ms, FOV = 220 × 220, matrix = 256 × 256). Eddy current and simple head motion correction was performed using FMRIB’s Diffusion Toolbox (FDT) within FMRIB’s Software Library (FSL version 5.0.9; Jenkinson et al., 2012).

### Current Modeling

**FIGURE 1:**
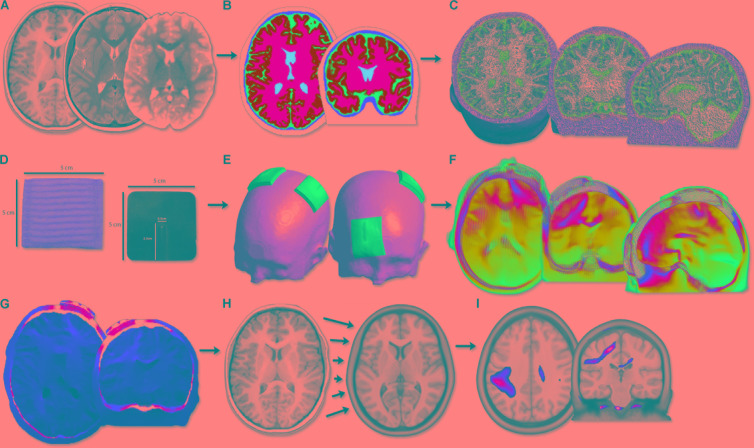
Image processing flow diagram. **(A)** Acquisition of T1, T2 anatomical and diffusion scans. **(B)** Segmentation of T1 anatomical image into five tissue types. **(C)** Calculation of head volume meshes composed of tetrahedral elements. **(D)** Modeling of 5 cm × 5 cm virtual tDCS electrodes. **(E)** Simulation of three typical tDCS montages aimed at motor learning (anodal, cathodal, bihemispheric). **(F)** Modeling of electric field strength across the brain. PeakEF was extracted for each montage, tissue type, and age group. **(G)** Conversion of EFMaps into NIfTI format by interpolating tetrahedral elements. **(H)** Normalization of NIfTI files into MNI template space using deformations calculated on T1 anatomy. **(I)** Voxel-based statistical analyses were performed using SPM12.

Transcranial direct-current stimulation current modeling was performed using the standard SimNIBS pipeline (**Figure 1**) (Thielscher et al., 2015). Briefly, the T1- and T2-weighted anatomical volumes were segmented into five tissue types using FSL and Freesurfer (version 5.3; Fischl et al., 2004) via the SimNIBS mri2mesh function. These segmentation surfaces corresponded to WM, GM, CSF, skull, and skin. Cerebellum and brainstem were categorized as WM. Segmentations were examined carefully slice-by-slice to ensure proper tissue classifications. Subsequently, tetrahedral volume mesh head models were created based on the segmentation surfaces using SimNIBS (Thielscher et al., 2015) and visualized using Gmsh (Geuzaine and Remacle, 2009).

Modeling of electric fields was performed through SimNIBS using FEM modeling on the generated volume meshes. Previously established conductivity values for each tissue type were used for FEM calculations (Opitz et al., 2015): WM [0.126 Siemans/meter (S/m)], GM (0.275 S/m), CSF (1.654 S/m), bone (0.010 S/m), and skin (0.465 S/m). Conductivity tensors were also calculated for the subset of participants with diffusion weighted sequences using a volume normalized approach, as described elsewhere (Opitz et al., 2011), to provide personalized anisotropic WM conductivity maps.

### Electrode Placement

**FIGURE 2:**
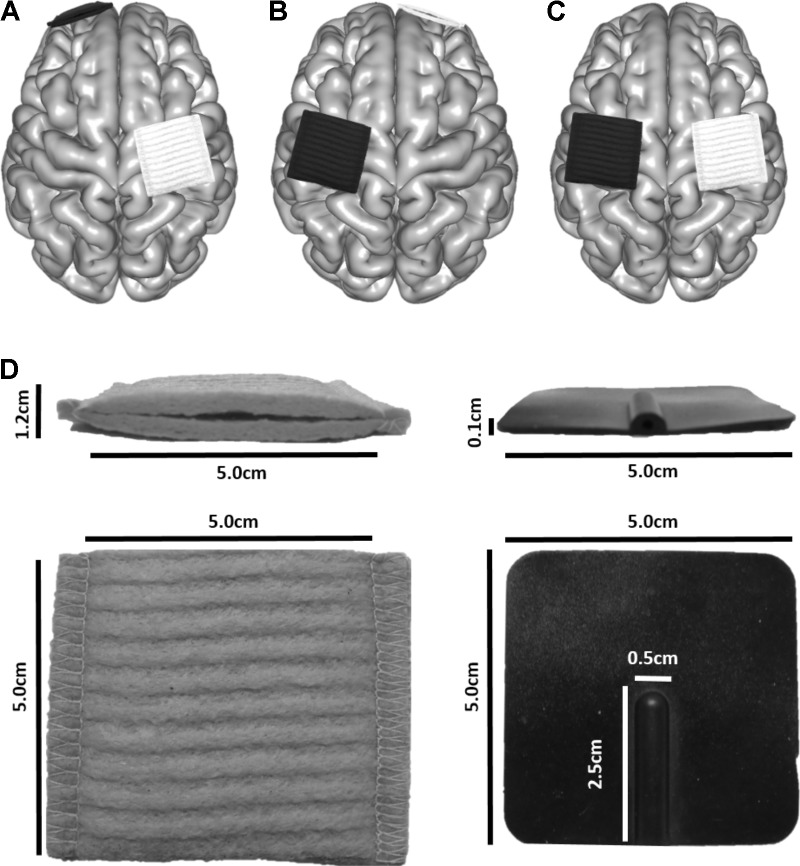
Electrode placement for three montages typically used in motor learning paradigms to enhance skill acquisition of the left hand. The white electrode depicts the anode, and the black the cathode. **(A)** M1_L_-SO_R,_ **(B)** SO_L_-M1_R,_ **(C)** M1_R_-M1_L_. **(D)** Electrode dimensions.

Virtual electrodes were generated using SimNIBS. Three tDCS montages applied in motor learning investigations in healthy adults and children (Vines et al., 2008; Reis et al., 2009; Ciechanski and Kirton, 2016a), and in stroke motor rehabilitation trials (Lindenberg et al., 2010; Kirton et al., 2017), were simulated: (A) anode directly above the hand area of right M1, cathode over the left supraorbit (M1_R_-SO_L_), (B) cathode over the right supraorbit, anode directly above the hand area of left M1 (SO_R_-M1_L_), (C) anode directly above the hand area of right M1 and cathode directly above the hand area of left M1 (M1_R_-M1_L_). These montages are typically used to enhance motor learning of the left hand and are illustrated in **Figures 2A–C**. The hand area of M1 (Yousry et al., 1997) was visually identified by a single investigator. For a subset of participants, the cathode was positioned directly above the hand area of right M1, with the anode over the left supraorbit (SO_L_-M1_R_). The purpose of this control was to ensure that polarity itself did not change electric field strength.

Electrodes were modeled to represent those produced by neuroConn (neuroConn GmbH; Ilmenau, Germany). Square sponges (5 cm × 5 cm) of 6 mm thickness were generated (**Figure 2D**). A rubber electrode of 1 mm thickness was enclosed in the sponge; the rubber electrode had a defined connector area of 2.5 cm × 0.5 cm, centered along the posterior aspect of the electrodes. Rubber electrode conductivity was set to 0.100 S/m, and saline-soaked sponges at 1.000 S/m. Current strength was set to 1 mA.

### Electric Field Modeling

Peak electric field (PeakEF) for each participant was identified in each tissue type for each tDCS montage using isotropic and anisotropic WM models. Subsequently, the final mesh representing the whole-brain electrical field model (EFMap) for each montage was converted to NIfTI format through the interpolation of electric field strength at the center of each voxel. This was done by smoothing surrounding tetrahedral mesh elements using a Gaussian weight function (based on distance from the center of the voxel) to create an EFMap with voxels of 1 mm^3^ resolution. To enable group comparisons, EFMaps for each participant were then converted to standardized Montreal Neurological Institute (MNI) space by calculating deformation fields based on each T1-weighted anatomical sequence using a mean template of 152 healthy adult individuals (MNI152) for comparison (Supplementary Figure S1). The same template was used for all participants to ensure that group statistics could be performed without systematic differences between age groups caused by normalization to different atlases. This normalization step was performed using Advanced Normalization Tools (ANTs; Avants et al., 2011) and the calculated warps were applied to each EFMap. Resulting warped whole-brain EFMaps were spatially smoothed using a 6 mm^3^ full-width half-maximum (FWHM) Gaussian kernel and were used in subsequent group comparisons. Spatial smoothing was performed to improve the normality of data for statistical analysis (Ashburner and Friston, 2000). For visualization, group mean EFMaps were created in SPM by averaging non-smoothed EFMaps for participants in each age group.

To specifically explore electric field strength corresponding to electrode placement, 30 mm-diameter spherical regions of interest (ROIs) were positioned within bilateral M1, ventromedial prefrontal cortex (VMPFC), and primary visual cortex (V1) on the MNI152 brain (Supplementary Figure S2). M1 ROI spheres were placed on the right and left hand-knob of the pre-central gyrus (Right M1: 36, -18, 60; Left M1: -36, -21, 61). VMPFC ROI spheres were placed in the frontal WM and GM (Right VMPFC: 18, 56, -5; Left VMPFC: -18, 56, -5). M1 and VMPFC ROI spheres corresponded to the cortical regions underlying the scalp electrodes. V1 ROI spheres were placed in occipital lobes (Right V1: -20, -86, -1; Left V1: -17, -89, 0) and served as reference ROIs to explore differences in electric field strength in areas spatially removed from the tDCS electrodes. Spheres were constrained to WM and GM (i.e., excluded CSF, skull, and skin). ROIs were superimposed on co-registered EFMaps for each participant and mean electric field strength (MeanEF) within each spherical ROI was extracted.

### Tissue Characteristics

For each participant, total cortical gray (GM_vol_) and white matter volumes (WM_vol_), estimated total intracranial volume (eTIV), and CSF volume (CSF_vol_) were calculated from the segmented Freesurfer volumes (all in mm^3^). Skull and CSF (extra-axial space) thickness for each participant was measured in locations corresponding to each tDCS electrode (i.e., left and right M1, right and left supraorbital area). Briefly, T1-weighted images were viewed in Freeview (Freesurfer’s image viewing tool) and skull, CSF, and GM binary masks were overlaid. A ruler tool was used to measure the distance (in mm) between various masks perpendicular to the surface of the brain. Skull thickness for supraorbital areas were measured using sagittal slices and were taken as the distance between the outside of the skull mask and the CSF mask. CSF thickness was measured as the difference between the outside of the CSF mask and the GM mask. The same procedure was performed for M1 areas using coronal slices. Participant skull thickness was averaged within each patient group to estimate group skull thickness under each tDCS electrode.

### Statistical Analyses

Distribution normality was assessed using Shapiro–Wilk tests. One-way analyses of variance (ANOVA) explored main effects of age (children, adolescents, adults) on tissue characteristics (GM_vol_, WM_vol_, eTIV, CSF_vol_, skull thickness), PeakEF and ROI EFs. Repeated-measures ANOVA (RM-ANOVA) explored differences between montages within tissue types. Holm–Sidak *post hoc* measures were employed to correct for multiple comparisons. A linear regression was used to explore the relationship between estimated GM_vol_, WM_vol_, eTIV, CSF_vol_, PeakEF, ROI EFs, and age. A linear regression was used to also explore the relationship between PeakEF and anatomical factors. Statistical analyses were performed using IBM SPSS (ver 20). Where relevant, values display mean (standard deviation). Statistical significance was evident when *p* < 0.05.

EFMaps for each participant were used in a group-level voxel-based statistical analysis in SPM (Statistical Parametric Mapping; UCL, Wellcome Trust) (Ashburner and Friston, 2000) to investigate differences in electric field strength across space. A one-way ANOVA followed by *post hoc* independent *t*-tests were used to investigate differences in electric field strength between participant age groups. Paired *t*-tests explored differences between using a single isotropic standard conductivity value for all WM versus using an individualized DWI-based anisotropic conductivity tensor. These comparisons were performed for each of three tDCS montages. For all analyses, a *p*-value of *p*_unc_ = 0.001 and a threshold cluster size of *k* > 100 voxels was used to determine significance.

## Results

### Population

Fifty-eight participants were recruited. Seven were subsequently excluded due to poor quality anatomical scans caused by excessive head motion or errors in modeling. The final study population consisted of 19 children, (median = 9.9 years, range = 6.5–12.7 years), 16 adolescents, (median = 16.8 years, range = 13.5–19.0 years), and 16 adults (median = 25.97 years, range = 20.9–43.0 years). Group demographic information is displayed in **Table 1**.

**Table 1:** Demographics and tissue characteristics.

Category	Participant group			Between group
		
Mean (SD) [range or %]	Children	Adolescents	Adults	
Age (years)	10.0 (1.8) [6.5–12.7]	16.3 (1.9) [13.5–19.0]	27.5 (6.3) [21–43]	*p* < 0.001
Sex [%]				
Male	*N* = 12 [63.2%]	*N* = 9 [56.3%]	*N* = 6 [37.5%]	*p* = 0.302
Female	*N* = 7 [36.8%]	*N* = 7 [43.8%]	*N* = 10 [62.5%]	
Total	*N* = 19	*N* = 16	*N* = 16	
GM_vol_ (mm^3^)	5.81 (0.5) [4.5–6.9]	5.35 (0.8) [4.0–6.8]	5.16 (0.5) [4.6–5.8]	*p* = 0.007
WM_vol_ (mm^3^)	4.29 (0.4) [3.7–5.4]	4.41 (0.8) [3.2–5.7]	4.71 (0.4) [4.2–5.7]	*p* = 0.080
eTIV (mm^3^)	1.56 (0.1) [1.3–1.8]	1.59 (0.2) [1.2–2.0]	1.58 (0.1) [1.4–1.8]	*p* = 0.920
CSF_vol_ (mm^3^)	49.4 (6.1) [37.3–58.9]	54.0 (3.0) [49.3–60.5]	47.35 (6.3) [35.8–56.4]	*p* = 0.011
Extra-axial space thickness (mm)				
Right M1	3.8 (1.1) [1.7–6.1]	5.1 (1.8) [2.1–8.7]	7.8 (2.4) [4.2–11.4]	*p* < 0.001
Left M1	4.2 (1.4) [2.2–7.4]	4.9 (1.7) [1.7–7.1]	7.7 (2.6) [3.1–11.6]	*p* < 0.001
Right supraorbit	4.4 (1.3) [1.9–7.5]	5.6 (2.2) [2.5–11.4]	5.7 (1.7) [3.3–8.6]	*p* = 0.056
Left supraorbit	5.1 (1.3) [2.7–8.0]	5.6 (1.9) [3.1–9.8]	5.7 (1.2) [4.2–7.9]	*p* = 0.441
Skull thickness (mm)				
Right M1	5.2 (1.0) [3.8–7.3]	7.1 (2.2) [4.6–11.2]	7.2 (1.3) [4.3–9.0]	*p* < 0.001
Left M1	5.1 (1.2) [3.1–7.8]	7.4 (2.0) [4.4–10.5]	7.1 (1.3) [4.5–9.0]	*p* < 0.001
Right supraorbit	6.7 (1.3) [4.5–9.3]	7.4 (1.7) [5.6–11.9]	8.8 (1.9) [5.7–12.6]	*p* < 0.002
Left supraorbit	7.1 (1.0) [5.4–9.4]	7.5 (1.8) [5.6–12.1]	9.2 (1.8) [6.4–12.8]	*p* < 0.001


*SD, standard deviation; GM_vol_, total cortical gray matter volume (×10^5^); WM_vol_, total cortical white matter volume (×10^5^); GM/WM ratio, GM_vol_/WM_vol_; eTIV, estimated intracortical volume (×10^6^); CSF_vol_, total cerebrospinal fluid volume. Note that some volumes are reported in scientific notation.*

### Tissue Characteristics

Tissue characteristics are reported in **Table 1**. GM_vol_ was significantly different among age groups [*F*(2,48) = 5.6, *p* = 0.007], where GM_vol_ was higher in children compared to adults (*p* = 0.007) with a trend toward differences between children and adolescents (*p* = 0.07). The linear regression revealed an inverse correlation between GM_vol_ and age (*R* = -0.46, *p* = 0.001). WM_vol_ also suggested possible, non-significant differences among age groups [*F*(2,48) = 2.7, *p* = 0.08]. The linear regression revealed a correlation between WM_vol_ and age (*R* = 0.29, *p* = 0.04).

CSF_vol_ was significantly different between age groups [*H*(2,48) = 9.0, *p* = 0.011]. where adolescents showed greater CSF_vol_ compared to adults (*p* = 0.006) but not children. No significant correlations with age were found for CSF_vol_. Extra-axial space thickness differed between age groups [*F*(2,48) = 11.7, *p* < 0.001]. *Post hoc* tests revealed that adults had thicker extra-axial spaces under left and right M1 electrode locations compared to both children and adolescents (both *p* < 0.001). Adolescents had thicker extra-axial spaces than children (*p* = 0.034). Extra-axial space thickness behind the supraorbits was not different among the three age groups. The linear regression demonstrated an association between extra-axial space thickness and age under both the left M1 (*R* = 0.67, *p* < 0.001) and right M1 electrodes (*R* = 0.70, *p* < 0.001). Skull thickness differed between age groups [*F*(2,48) = 11.1, *p* < 0.001], where children had thinner skulls than adolescents and adults under left and right M1 electrode locations (both *p* < 0.001). There was no difference in skull thickness between adolescents and adults under left and right M1 electrode locations (both *p* > 0.865). Adults has significantly thicker skulls behind the supraorbital electrodes compared to children (both *p* < 0.001) and adolescents (both *p* < 0.019). There was no difference in skull thickness behind the supraorbital electrodes when comparing children and adolescents (both *p* > 0.275). The linear regression demonstrated a significant association between skull thickness and age under M1 electrodes (both *r* > 0.345, *p* < 0.013) and behind supraorbital electrode (both *r* > 0.467, *p* < 0.001), where thickness increased with age. No relationship between age and eTIV was observed.

### Peak Electric Fields

#### M1_R_-SO_L_ Montage

**FIGURE 3:**
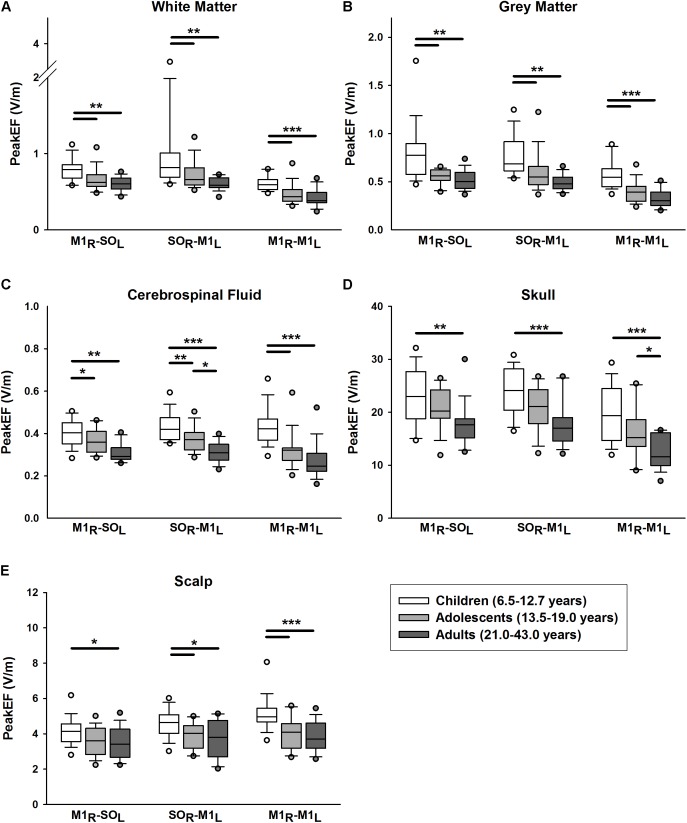
Peak electric fields (PeakEF) induced by anodal, cathodal, and bihemispheric tDCS montages in children (white), adolescents (light gray) and (dark gray) by tissue type as follows: **(A)** white matter, **(B)** gray matter, **(C)** cerebrospinal fluid, **(D)** skull, and **(E**) scalp. ^∗^*p* < 0.05, ^∗∗^*p* < 0.01, ^∗∗∗^*p* < 0.001.

PeakEF induced by tDCS in five tissue types (WM, GM, CSF, skull, and scalp) for all tDCS montages are summarized in **Figure 3**. M1_R_-SO_L_ tDCS induced significantly stronger PeakEF in children than adults in all tissue types (all *p* < 0.027). Children also displayed stronger PeakEF in WM, GM, and CSF, compared to adolescents (all *p* < 0.017). PeakEF was negatively correlated with age in all tissues (**Table 2**) suggesting weaker PeakEF with increasing age. When electrode polarity was reversed (SO_L_-M1_R_), there was no difference in PeakEF in all tissue types.

**Table 2:** Correlations of PeakEF across tissue types.

			Anatomical factor		
			
Tissue type	Montage	Age	Skull thickness	CSF_Vol_	eTIV
WM	Anodal	-0.392^∗∗^	-0.493^∗∗∗^	-0.167	-0.036
	Cathodal	-0.345^∗^	-0.337^∗^	-0.296^∗^	0.024
	Bihemispheric	-0.456^∗∗∗^	-0.597^∗∗∗^	-0.240	0.182
GM	Anodal	-0.427^∗∗^	-0.415^∗∗^	-0.242	0.04
	Cathodal	-0.420^∗∗^	-0.483^∗∗∗^	-0.218	-0.157
	Bihemispheric	-0.524^∗∗∗^	-0.613^∗∗∗^	-0.179	0.209
CSF	Anodal	-0.505^∗∗∗^	-0.658^∗∗∗^	-0.018	-
	Cathodal	-0.567^∗∗∗^	-0.681^∗∗∗^	0.071	-
	Bihemispheric	-0.506^∗∗∗^	-0.675^∗∗∗^	-0.097	-
Skull	Anodal	-0.495^∗∗^	-0.468^∗∗∗^	-	-
	Cathodal	-0.437^∗∗^	-0.663^∗∗∗^	-	-
	Bihemispheric	-0.543^∗∗∗^	-0.619^∗∗∗^	-	-
Scalp	Anodal	-0.310^∗^	-	-	-
	Cathodal	-0.307^∗^	-	-	-
	Bihemispheric	-0.357^∗^	-	-	-


*CSF, cerebrospinal fluid; eTIV, estimated total intracranial volume; GM, gray matter; WM, white matter. ^∗^*p* < 0.05, ^∗∗^*p* < 0.01, ^∗∗∗^*p* < 0.001.*

#### SO_R_-M1_L_ Montage

Similar patterns were observed for SO_R_-M1_L_ tDCS which induced stronger PeakEF in children compared to adults in all tissue types (all *p* < 0.015). Children also displayed stronger PeakEF in all tissue types (all *p* < 0.044) excluding the skull (*p* = 0.056), compared to adolescents. Additionally, compared to adults, adolescents displayed stronger PeakEF in CSF (*p* = 0.010). PeakEF in all tissue types was negatively correlated with age (**Table 2**), suggesting weaker PeakEF with increasing age.

#### M1_R_-M1_L_ Montage

M1_R_-M1_L_ tDCS also induced stronger PeakEF in all tissue in children compared to both adolescents (all *p* < 0.001) and adults (WM, GM, CSP, and scalp, *p* < 0.001; skull, *p* = 0.032). PeakEF was negatively correlated with age (**Table 2**), suggesting weaker PeakEF with increasing age.

The linear regression for factors correlated with age, including skull thickness, extra-axial space thickness, and GM-WM ratio, revealed correlations with PeakEF. PeakEF decreased as both skull and extra-axial space thickness increased (**Table 2**).

PeakEF was also compared across tDCS montages. In WM, both M1_R_-SO_L_ (*t* = 3.487, *p* = 0.001) and SO_R_-M1_L_ tDCS (*t* = 5.337, *p* < 0.001) induced stronger PeakEF compared to bihemispheric tDCS. No difference in PeakEF was seen between M1_R_-SO_L_ and SO_R_-M1_L_ montages, although trends toward higher PeakEF induced by SO_R_-M1_L_ tDCS were suggested (*t* = 1.850, *p* = 0.067). Interaction effects suggested that both M1_R_-SO_L_ (*t* = 2.030, *p* = 0.045) and SO_R_-M1_L_ montages (*t* = 4.885, *p* < 0.001) induced higher PeakEF than M1_R_-M1_L_ montages in children. SO_R_-M1_L_ induced higher PeakEF compared to M1_R_-SO_L_ montages (*t* = 2.825, *p* = 0.012). Adults demonstrated no difference in induced PeakEF between montages (all *t* < 1.975, all *p* > 0.100).

In GM, M1_R_-SO_L_ (*t* = 6.899, *p* < 0.001) and SO_R_-M1_L_ tDCS (*t* = 6.824, *p* < 0.001) induced stronger PeakEF compared to M1_R_-M1_L_ tDCS. No difference in PeakEF was seen between M1_R_-SO_L_ and SO_R_-M1_L_ montages (*t* = 0.075, *p* = 0.941). Interaction effects suggest that in all age groups M1_R_-SO_L_ (all *t* > 3.010, all *p* < 0.008) and SO_R_-M1_L_ tDCS (all *t* > 3.549, all *p* < 0.002) induced higher PeakEF than M1_R_-M1_L_ montages. In all age groups there were no differences in PeakEF between M1_R_-SO_L_ and SO_R_-M1_L_ montages (all *t* < 0.808, all *p* > 0.421).

In CSF, SO_R_-M1_L_ tDCS induced stronger PeakEF compared to M1_R_-M1_L_ tDCS (*t* = 3.049, *p* = 0.009). Interaction effects suggest that only adolescents showed this difference between SO_R_-M1_L_ and bihemispheric tDCS (*t* = 2.780, *p* = 0.020), although possible effects were observed in adults (*t* = 2.397, *p* = 0.054). In the skull, both M1_R_-SO_L_ (*t* = 7.188, *p* < 0.001) and SO_R_-M1_L_ tDCS (*t* = 7.824, *p* < 0.001) induced higher PeakEF compared to M1_R_-M1_L_ tDCS. These differences were evident in all age groups (all *t* > 3.527, all *p* < 0.002). In the scalp, montage-specific differences in PeakEF were observed (M1_R_-SO_L_ vs. SO_R_-M1_L_, *t* = 2.366, *p* = 0.040; M1_R_-SO_L_ vs. M1_R_-M1_L_, *t* = 4.710, *p* < 0.001; SO_R_-M1_L_ vs. M1_R_-M1_L_, *t* = 2.345, *p* = 0.021). M1_R_-M1_L_ montages induced the strongest PeakEF, followed by SO_R_-M1_L_ montages, and lastly M1_R_-SO_L_ tDCS. Analysis of interactions revealed that only in children did PeakEF differ between montages (all *t* < 1.874, all *p* < 0.050). No significant differences in PeakEF were seen between montages in adolescents (all *t* < 1.868, all *p* > 0.182) or adults (all *t* < 1.926, all *p* > 0.162).

### Whole Brain Electric Field Maps

#### M1_R_-SO_L_ Montage

**FIGURE 4:**
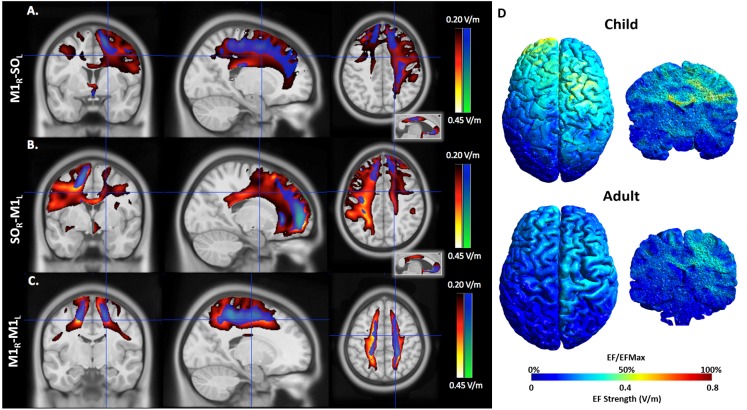
An illustration of electric field maps induced by **(A)** anodal tDCS (right M1), **(B)** cathodal tDCS (left M1), and **(C)** bihemispheric tDCS montages. Shown are group mean electric field maps for children (red) and adults (blue), in the range of 0.20–0.45 V/m, overlaid on three orientations (axial, coronal, sagittal) of an MNI template brain. **(D)** Example of electric fields induced by anodal tDCS of right primary cortex in a 6.5 (child, top) and 43.0-year-old (adult, bottom).

**FIGURE 5:**
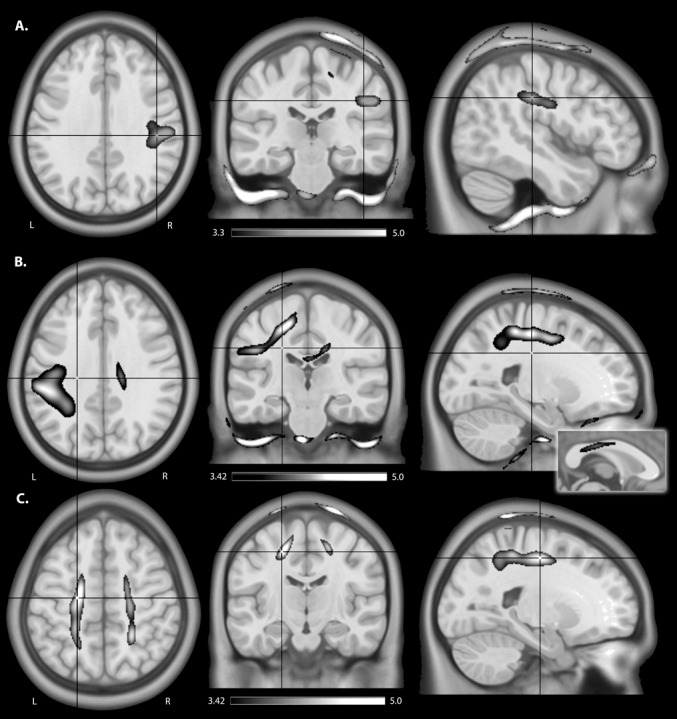
An illustration of areas of higher estimated electric field strength in children compared to adults using **(A)** isotropic WM maps for anodal tDCS, **(B)** anisotropic WM tensors for cathodal, and anisotropic WM tensors for **(C)** bihemispheric tDCS montages. Shown are statistical *T*-score heat maps overlaid on three orientations (axial, coronal, sagittal) of an MNI template brain [significance threshold: *T*(33) > 3.3, *p* < 0.001]. These maps display statistical *T*-score heat maps [significance threshold *T*(27) > 3.42, *p* < 0.001] overlaid on an MNI template brain. The inset for **(B)** on the midline sagittal slice illustrates higher estimated EF in the transcallosal motor fibers of the corpus callosum for children compared to adults.

EFMaps are illustrated in **Figure 4**. Children demonstrated a wider spread of induced electric fields by M1_R_-SO_L_ tDCS compared to adults (**Figure 4A**) and teenagers. In children, cortical regions underlying the anode, stimulated in the range of 0.20–0.45 V/m, extended continuously from the cortical surface to deeper areas including the internal capsule. Additional electric fields were observed in the corpus callosum and the contralateral pre-central gyrus including underlying WM tracts. Induced electric fields in adolescents (not shown) appeared similar to those of adults, primarily limited to the WM underlying the pre-central gyrus and frontal lobe. Voxel-based statistical analyses indicated several areas of significantly stronger electric fields for children compared to adults (**Figure 5A**). Differences were seen in the right superior frontal/parietal WM underlying the pre/post-central gyrus M1 anode [*T*(33) = 3.87, *p* < 0.001, *k* = 1908 voxels, peak MNI 10, -33, 63] and in more inferior and lateral areas [*T*(33) = 4.43, *p* < 0.001, *k* = 4927 voxels, peak MNI 54, -24, 32]. Differences were also seen in the left superior frontal gyrus, corresponding to the placement of the supraorbital frontal cathode [*T*(33) = 3.85, *p* < 0.001, *k* = 927 voxels, peak MNI -20, 59, -2].

EFMaps were also calculated using each participants’ diffusion-weighted sequences to estimate a personalized anisotropic conductivity tensor for modeling electric current flow through WM. Voxel-based statistical analyses indicated no areas of significantly higher electric field strength for children compared to adults for the M1_R_-SO_L_ montage.

#### SO_R_-M1_L_ Montage

As with the M1_R_-SO_L_ montage, children demonstrated a more widespread induction of electric fields compared to both teenagers and adults with cathodal stimulation montages (**Figure 4B**). In children, cortical regions underlying the cathode appeared more widely stimulated compared to adults with induction continuously observed from the cortical surface to deeper areas including the internal capsule. In children, but not adults (and to a lesser extent in adolescents), electric fields were induced in the corpus callosum and contralateral pre-central gyrus. Current was suggested in both the right and left frontal lobes in children. For the SO_R_-M1_L_ montage, stronger electric fields were seen in children compared to adults in left superior frontal/parietal WM areas underlying the placement of the M1 cathode [*T*(33) = 3.67, *p* < 0.001, *k* = 896 voxels, peak MNI -19, -38, 56] and more inferior and lateral WM areas [*T*(33) = 3.86, *p* < 0.001, *k* = 1243 voxels, peak MNI -59, -28, 33]. Areas of higher electric field strength were also seen approximating the right supraorbital anode in the superior frontal gyrus [*T*(33) = 4.02, *p* < 0.001, *k* = 647 voxels, peak MNI 18, 60, -5].

When anisotropic WM tensors were used in the current model, a similar but larger age-dependent pattern of electric field strength was seen (**Figure 5B**). Higher field strength was observed for children over adults in the left superior frontal/parietal WM [*T*(27) = 4.89, *p* < 0.001, *k* = 25811 voxels, peak MNI -20, -38, 55] underlying the M1 cathode. Additional areas of higher EF were seen in the midportion of the corpus callosum WM [*T*(27) = 4.13, *p* < 0.001, *k* = 2990 voxels, peak MNI 16, -23, 32] possibly corresponding to transcallosal motor and sensory fibers (Hofer and Frahm, 2006) and in the supraorbital frontal areas approximating the anode [*T*(27) = 4.58, *p* < 0.001, *k* = 1185 voxels, MNI 18, 59, -5].

#### M1_R_-M1_L_ Montage

M1_R_-M1_L_ montages showed less spatial differences between children and adults compared to the unilateral montages described above (**Figure 5C**). Induction of electric fields was primarily limited to GM and WM underlying the anode and cathode. However, in children electric fields appeared to spread inferiorly along WM while in adults, fields were more constrained to superior cortical regions. The M1_R_-M1_L_ montage also showed higher electric field strength for children over adults in two main areas corresponding to the placement of the electrodes; the right anode [*T*(33) = 4.55, *p* < 0.001, *k* = 2275 voxels, peak MNI 15, -43, 79] and left cathode over the pre/post central gyrus [*T*(33) = 4.32, *p* < 0.001, *k* = 872 voxels, peak MNI -20, -43, 77]. Using anisotropic WM tensors in the model suggested similar but larger age-dependent difference in electric field strength in the WM underlying the right M1 anode [*T*(27) = 4.64, *p* < 0.001, *k* = 6671 voxels, peak MNI 24, -46, 49] and the left M1 cathode [*T*(27) = 5.06, *p* < 0.001, *k* = 10774 voxels, peak MNI -19, -12, 52].

### Comparison Between WM Conductivity Calculations

Paired *t*-tests of estimated electric field strength between models using isotropic and anisotropic WM conductivity tensors showed large differences. Specifically, for the SO_R_-M1_L_ montage, using individualized anisotropic WM tensors suggested stronger electric field strength for whole brain WM in all age groups particularly in the frontal WM and corpus callosum of children (Supplementary Figure S3). The anisotropic M1_R_-M1_L_ montage also suggested higher electric field strength in whole brain WM for both children and adolescents, extending into the temporal lobe (Supplementary Figure S3).

### ROI Analysis

MeanEF was calculated within the hand-knob region of M1 (**Table 3**). In right M1, M1_R_-SO_L_ tDCS induced significantly stronger MeanEF in children compared to adolescents and adults (*p* < 0.001). There was no difference in MeanEF strength between adults and adolescents (*p* = 0.244). Similar trends were seen within the left VMPFC, however, MeanEF was higher than in right M1 (all age groups, *p* < 0.001). SO_R_-M1_L_ tDCS induced significantly higher MeanEF strength in children in left M1 compared to adolescents and adults (both *p* < 0.001). There was no difference in MeanEF strength between adults and adolescents (*p* = 0.516). Similar trends were seen within the right VMPFC, where MeanEF was higher than in left M1 (all age groups, *p* < 0.001). M1_R_-M1_L_ tDCS induced stronger MeanEF in children in left M1 compared to adolescents (*p* = 0.005) and adults (*p* < 0.001). MeanEF in left M1 was also higher in adolescents than adults (*p* = 0.049). Similarly, in right M1 the MeanEF in children was higher than adolescents (*p* = 0.003) and adults (*p* < 0.001). Adolescents also showed higher MeanEF than adults (*p* = 0.030).

**Table 3:** Average electric field strengths.

		Average electric field strength (V/m)			Between group *post hoc*		
			
ROI	Montage	1. Children	2. Adolescents	3. Adults	1 vs. 2	1 vs. 3	2 vs. 3
M1_L_	M1_R_-SO_L_	0.144 (0.020)	0.116 (0.017)	0.108 (0.019)	^∗∗∗^	^∗∗∗^	n.s.
	SO_R_-M1_L_	0.200 (0.036)	0.147 (0.040)	0.138 (0.029)	^∗∗∗^	^∗∗∗^	n.s.
	M1_R_-M1_L_	0.168 (0.027)	0.139 (0.025)	0.120 (0.028)	^∗∗^	^∗∗∗^	^∗^
M1_R_	M1_R_-SO_L_	0.194 (0.027)	0.147 (0.036)	0.134 (0.028)	^∗∗∗^	^∗∗∗^	n.s.
	O_R_-M1_L_	0.149 (0.020)	0.117 (0.025)	0.106 (0.019)	^∗∗∗^	^∗∗∗^	n.s.
	M1_R_-M1_L_	0.168 (0.021)	0.138 (0.028)	0.118 (0.027)	^∗∗^	^∗∗∗^	^∗^
VMPFC_L_	M1_R_-SO_L_	0.249 (0.043)	0.215 (0.028)	0.199 (0.028)	^∗^	^∗∗∗^	n.s.
	SO_R_-M1_L_	0.157 (0.027)	0.133 (0.028)	0.130 (0.016)	^∗^	^∗∗^	n.s.
	M1_R_-M1_L_	0.050 (0.008)	0.054 (0.011)	0.047 (0.012)	n.s.	n.s.	n.s.
VMPFC_R_	M1_R_-SO_L_	0.154 (0.025)	0.145 (0.039)	0.130 (0.014)	^∗^	n.s.	n.s.
	SO_R_-M1_L_	0.246 (0.039)	0.197 (0.041)	0.182 (0.031)	^∗∗∗^	^∗∗∗^	n.s.
	M1_R_-M1_L_	0.049 (0.006)	0.057 (0.016)	0.050 (0.017)	n.s.	n.s.	n.s.
V1_L_	M1_R_-SO_L_	0.077 (0.008)	0.065 (0.010)	0.060 (0.008)	^∗∗∗^	^∗∗∗^	n.s.
	SO_R_-M1_L_	0.087 (0.010)	0.072 (0.017)	0.067 (0.012)	^∗∗∗^	^∗∗∗^	n.s.
	M1_R_-M1_L_	0.053 (0.007)	0.052 (0.010)	0.047 (0.010)	n.s.	n.s.	n.s.
V1_R_	M1_R_-SO_L_	0.089 (0.010)	0.076 (0.014)	0.069 (0.011)	^∗∗^	^∗∗∗^	n.s.
	SO_R_-M1_L_	0.079 (0.010)	0.064 (0.016)	0.056 (0.010)	^∗∗^	^∗∗∗^	n.s.
	M1_R_-M1_L_	0.055 (0.009)	0.053 (0.008)	0.046 (0.001)	n.s.	^∗^	n.s.


*L, left; M1, primary motor cortex; R, right; SO, supraorbital area; V1, primary visual cortex; VMPFC, ventromedial prefrontal cortex. Shown are group means (standard deviation). ^∗^*p* < 0.05, ^∗∗^*p* < 0.01, ^∗∗∗^*p* < 0.001, n.s. *p* > 0.05.*

## Discussion

Here, we modeled tDCS-induced electric fields through the brain in groups of typically developing children, adolescents, and adults using individualized MRI anatomy. In this cross-sectional study, we have demonstrated that children may experience stronger and more widespread electric fields compared to adults. Our results suggest that these differences may be associated with age-related differences in skull and extra-axial space thickness as well as developmental changes occurring in GM and WM. Individualized current modeling may therefore be a valuable tool for personalizing effective doses of tDCS in future pediatric clinical trials.

To our knowledge, this is the largest tDCS modeling study using a pediatric sample to date. We found significantly higher peak electric field strength, higher mean electric field strength, and more expansive electric field spread for children compared to adults using individualized MRI anatomy for all tDCS montages. These findings may relate to the significantly thinner skulls of children compared to adults. Skull is much less conductive than other tissue, and therefore reduces the transmission of current generated by tDCS (Opitz et al., 2015). Our results suggest that the thinner skulls of children may reduce blockage of current, resulting in more current reaching the underlying neuronal tissue, inducing stronger electric fields. This finding is supported by strong correlations between skull thickness and PeakEF in GM, WM, CSF, and skull, for all montages. In addition to changes in skull thickness, conductivity of the skull may also change with age. Conductivity measures suggest that the developing immature skull may be more conductive than that of older individuals (Wendel and Malmivuo, 2006). Here, we held the conductivity of the skull constant, and therefore if age-appropriate conductivity values were used we would expect even larger differences in PeakEF between children and adults. We acknowledge that this is a limitation of the current study.

In our cohort, we found that skull thickness of superior frontal-parietal bones showed thickening between childhood and adolescence, stagnating between adolescence and adulthood. Conversely, supraorbital skull areas show thickening between adolescence and adulthood, but not childhood and adolescence. In combination with the smaller extra-axial CSF spaces seen in children, this implies a shorter scalp-brain distance and reduced current shunting, which would be expected to increase induced electric fields under the electrode. Children younger than that of our cross-section (age < 6.5 years) have even thinner skulls (Li et al., 2015) and shorter scalp-brain distances, warranting further caution as electric fields may be even stronger. This finding also has implications for patients who have skull malformations or post-surgical patients that have undergone craniotomy where the removal of regions of skull may lead to significantly stronger electric field in brain tissue. Large differences in induced electric field have previously been modeled in these patients (Datta et al., 2010). Overall, application of tDCS across the thinner skulls of children may result in substantially stronger electric fields than in adults. However, it should be carefully noted that even much higher currents than those estimated here still fall within an order of magnitude lower than the agreed upon lower limit for risk of possible harm (Liebetanz et al., 2009; Bikson et al., 2016).

Brain development before the age of 20 is a time of significant change in both WM and GM. Typically, GM volumes increase during early childhood then decrease after age ∼10-12 (Giedd et al., 1999; Sowell et al., 2004; Shaw et al., 2008; Ducharme et al., 2016) as normal synaptic pruning processes occur (Huttenlocher, 1979; Huttenlocher et al., 1982; Brain Development Cooperative Group, 2012). The exact timing and trajectories of these dynamic changes in GM vary across brain structures and with gender, and continue to be investigated as improved MRI technologies are developed to non-invasively examine typical development across childhood. WM volume, by contrast, typically shows a different pattern, increasing during early childhood and adolescence and then stabilizing in early adulthood (Giedd et al., 1999; Groeschel et al., 2010). Specifically, MRI diffusion measures of WM microstructure, such as fractional anisotropy, mean diffusivity, neurite orientation dispersion index and neurite density index all change during childhood and adolescence (Lebel et al., 2008; Giorgio et al., 2010; Chang et al., 2015). These imaging biomarkers presumably reflect multiple processes that may include extensive myelination processes, changes in axonal packing/density and axonal membranes as well as increases in axonal diameter (Beaulieu, 2002; Paus, 2005). GM and WM development do not occur in isolation and are inter-related. For example, it has been proposed that the apparent decrease in GM volume in adolescence is not due to GM volume reduction *per se*, but rather to increasing myelination of WM that changes the MR signal at the GM-WM boundary (Groeschel et al., 2010). The resulting GM–WM ratio therefore decreases throughout adolescence into adulthood. Our results are potentially consistent with these dynamic developmental changes, suggesting factors such as decreasing GM volumes, increasing WM volumes and decreasing GM–WM ratios may relate to age-specific differences in tDCS current models. Despite extensive changes in brain tissue into adulthood, total intracranial volume is thought to somewhat stabilize by the age of 5 years (Brain Development Cooperative Group, 2012), consistent with our findings of no systematic relationship between age and estimated intracranial volume in our slightly older sample (aged > 6 years). Our findings support the importance of the developmental maturation of brain tissue itself in designing age-specific tDCS applications.

In terms of tissue characteristics, lower WM conductivity resulted in higher peak electric fields, perhaps in part due to current being less efficiently conducted (Opitz et al., 2015) and therefore more focally intense. Thus, developmental increases in conductivity may be consistent with our finding of induction of stronger WM peak electric fields in children over adults. Furthermore, we also observed large differences in electric field strength using volume normalized anisotropic WM tensors in our current modeling calculations compared to using a common scalar value for all participants. That this additional data had such an impact on our models suggests valuable utility in including individualized diffusion-weighted sequences for each participant when modeling current flow in pediatric populations. This is may be especially true in samples of children with brain lesions or malformations. Since electric fields are particularly sensitive to changes in conductance of different tissue types (i.e., when moving from CSF to GM to WM), care should be taken to understand tDCS-induced electric field strengths in young children with abnormal brain structure undergoing tDCS interventions. This is clinically relevant as most early clinical trials of tDCS in children have appropriately focused on cerebral palsy and its associated disability where the majority of participants have structural brain lesions (Kirton, 2017). Current modeling studies of specific brain diseases in children are required to better understand the effects of such lesions if precise, personalized solutions to tDCS therapeutics are to be realized.

M1_R_-SO_L_ (representative of an anodal tDCS montage) and SO_R_-M1_L_ (representative of a cathodal tDCS montage) typically induced comparable peak electric fields in GM, WM, CSF, and skull. Interestingly, we found that SO_R_-M1_L_ induced stronger peak electric fields on the scalp compared to M1_R_-SO_L_. These differences were not polarity specific, as M1_R_-SO_L_ and SO_L_-M1_R_ montages induced identical PeakEF in all tissues, but was likely related to minute anatomical differences underlying the electrodes. Our previous findings indicate that sensations such as itching and tingling are more commonly reported with cathodal compared to anodal stimulation in children (Ciechanski and Kirton, 2016a). This observation is supported by our current modeling findings, suggesting that the differences in sensations between these two montages may be related to the strength of induced electric fields. M1_R_-M1_L_ (representative of a bihemispheric tDCS montage) induced even stronger electric fields across the scalp, therefore it is possible that bihemispheric tDCS induces stronger sensations than anodal or cathodal montages, although direct comparisons have not been made in pediatric populations. That our previous study also suggested that children are more likely to report sensations as moderate or severe as compared to adolescents is also consistent with our current modeling results of stronger electric fields across the scalp in this age group.

Our current modeling findings here may also help indirectly explain early descriptions suggesting unique neurophysiological effects of tDCS in children. Cathodal tDCS displays non-linear changes in cortical excitability in adults (Batsikadze et al., 2013) where a 1 mA current (35 cm^2^ electrodes) may reduce cortical excitability while a 2 mA current increases excitability. In children, similar effects have been described with weaker currents, where a 0.5 mA and 1 mA current decreased and increased cortical excitability, respectively (Moliadze et al., 2015). Here, we demonstrate that mean electric field strength induced in M1 is stronger in children than adults. Applying a 1 mA current therefore induces different strengths of electric fields in children and adults, where a 1 mA current induces electric fields in children that are comparable to those that a 2 mA current induces in adults. Therefore, the strength of induced electric fields in M1 may dictate neurophysiological effects, as opposed to the current (density) applied. There is clearly a need for further neurophysiological studies of tDCS effects in children to better define these apparent differences. It is important to note that while our study only applied 1 mA tDCS, computational models scale linearly, and therefore the relative electric field differences between adults and children apply across all stimulation intensities.

Our current modeling findings hold implications for an improved understanding of the effects of tDCS on motor learning in children. tDCS enhances motor learning in both healthy adults and children, often producing large effect sizes and sustained effects (Reis and Fritsch, 2011; Ciechanski and Kirton, 2016a; Buch et al., 2017). We recently demonstrated that both anodal and cathodal montages safely enhance motor learning in healthy children. Our findings suggest that the effects of tDCS on motor skill acquisition in children are not identical to those in adults (Ciechanski and Kirton, 2016a). Here, our models suggest that current spread in children may extend as far as the contralateral hemisphere. Significant current flow through other premotor structures likely to be involved in motor learning and control may also be relevant in younger subjects. In adults, the spread of current may be more restricted to the stimulated M1, rather than including these more distant regions of the motor network.

Our study has limitations. Current modeling calculations are based on many assumptions such as previously established tissue and skull conductivities. While tissue conductivities were not directly measured in this study, they were reasonable approximations based on previous literature (Opitz et al., 2015). Recently, FEM predictions have been validated *in vivo* (Huang et al., 2017). While this validation study was performed using different modeling software, the basic principles behind these methodologies was consistent with those we employed, suggesting that our electric field predictions may be valid as well. Next, tissue segmentations were performed automatically and were therefore limited by the quality, resolution and contrast of the T1 and T2 anatomical scans. Anatomical scans were kept identical to standardize sequence collection across patient groups, however, participant weight was used for energy deposition calculations by the scanner which may have resulted in slight differences between groups. To ensure best possible tissue segmentation, individual scans were reviewed, slice by slice. Furthermore, we were only equipped to segment five tissue types, however, other tissues (such as eyeballs and air) may not be as influential in current modeling. As with any normalization step, a certain degree of distortion occurs when warping an image from native participant space to standard MNI space. We used a well-established, reliable normalization tool (ANTS) to calculate the deformation fields (Klein et al., 2009; Avants et al., 2011) and visually inspected image outputs overlaid on the MNI template at every step to ensure that normalization was successful. In the voxel-based analysis, spatial smoothing of each EFmap was performed using a 3-dimensional Gaussian kernel. Smoothing may lead to concerns that strong electric fields in skull may be smoothed into the parenchyma. Regions with significantly stronger stimulation between children and adults were typically located deeper in the white matter, outside the range of the Gaussian kernel, therefore it is unlikely these significant regions were attributed only to smoothing. Furthermore, if smoothing caused “spillover” of electric fields, we would expect to see areas of significantly stronger electric fields in the CSF and surface of the cortex, which we did not. No corrections were made for head motion in the anatomical scans possibly leading to additional variability between patient groups given that children are more likely to move during scanning. Simple head motion corrections between diffusion volumes were performed for the DTI sequence using FSL’s FDT Eddy correct function. We also did not correct for multiple comparisons using stringent family-wise error corrections during statistical analysis. Rather, we used a conservative significance threshold of *p* < 0.001 and a minimum cluster size of 100 voxels. Spurious false positive results would be unlikely to occur in a cluster of this size. Our sample size was sufficiently large to detect differences in electric field strength among groups, however, may not have been powerful enough to model more complex relationships (cubic, quadratic) among variables. Finally, this was a cross-sectional sample and more difficult, longitudinal designs to directly quantify developmental changes within subjects over time for all variables would be more powerful and indicative of true developmental trajectories.

## Conclusion

In conclusion, we demonstrate that children, adolescents and adults experience differences in the strength and spread of tDCS-induced electric fields. Electric field strength induced M1-targeting tDCS montages result in stronger electric fields in children compared to both adolescents and adults. With respect to the spread of induced electric fields, tDCS stimulates more widespread areas of the brain compared to adolescents and adults. While adolescents and adults show relatively similar stimulation patterns, children may experience stronger and more widespread current compared to adults, signifying substantial variability in pediatric populations. Our findings warrant safety monitoring for tDCS application in pediatrics, and future investigations might use current modeling techniques to plan individualized treatment for subsequent clinical trials.

## Author Contributions

PC and HC contributed to study design, data processing, data analysis, statistical analysis, and manuscript preparation. SY contributed to data analysis and manuscript preparation. AK contributed to study design, manuscript preparation, and obtaining funding.

## Conflict of Interest Statement

The authors declare that the research was conducted in the absence of any commercial or financial relationships that could be construed as a potential conflict of interest.

## References

[B1] Ashburner J. Friston K. J. (2000). Voxel-based morphometry–the methods. *Neuroimage* 11 805–821. 10.1006/nimg.2000.0582 10860804

[B2] Avants B. B. Tustison N. J. Song G. Cook P. A. Klein A. Gee J. C. (2011). A reproducible evaluation of ANTs similarity metric performance in brain image registration. *Neuroimage* 54 2033–2044. 10.1016/j.neuroimage.2010.09.025 20851191 PMC3065962

[B3] Batsikadze G. Moliadze V. Paulus W. Kuo M.-F. Nitsche M. A. (2013). Partially non-linear stimulation intensity-dependent effects of direct current stimulation on motor cortex excitability in humans. *J. Physiol.* 591 1987–2000. 10.1113/jphysiol.2012.249730 23339180 PMC3624864

[B4] Beaulieu C. (2002). The basis of anisotropic water diffusion in the nervous system - a technical review. *NMR Biomed.* 15 435–455. 10.1002/nbm.782 12489094

[B5] Bikson M. Grossman P. Thomas C. Zannou A. L. Jiang J. Adnan T. (2016). Safety of transcranial direct current stimulation: evidence based update 2016. *Brain Stimul.* 9 641–661. 10.1016/j.brs.2016.06.004 27372845 PMC5007190

[B6] Brain Development Cooperative Group (2012). Total and regional brain volumes in a population-based normative sample from 4 to 18 years: the NIH MRI study of normal brain development. *Cereb. Cortex* 22 1–2. 10.1093/cercor/bhr018 21613470 PMC3236790

[B7] Buch E. R. Santarnecchi E. Antal A. Born J. Celnik P. A. Classen J. (2017). Effects of tDCS on motor learning and memory formation: a consensus and critical position paper. *Clin. Neurophysiol.* 128 589–603. 10.1016/j.clinph.2017.01.004 28231477

[B8] Chang Y. S. Owen J. P. Pojman N. J. Thieu T. Bukshpun P. Wakahiro M. L. J. (2015). White matter changes of neurite density and fiber orientation dispersion during human brain maturation. *PLoS One* 10:e0123656. 10.1371/journal.pone.0123656 26115451 PMC4482659

[B9] Ciechanski P. Kirton A. (2016a). Transcranial direct-current stimulation can enhance motor learning in children. *Cereb. Cortex* 27 2758–2767. 10.1093/cercor/bhw114 27166171

[B10] Ciechanski P. Kirton A. (2016b). “Transcranial direct-current stimulation (tDCS): principles and emerging applications in children,” in *Pediatric Brain Stimulation: Mapping and Modulating the Developing Brain*, 1st Edn, eds Kirton A. Gilbert D. (New York City, NY: Elsevier), 475.

[B11] Datta A. Bansal V. Diaz J. Patel J. Reato D. Bikson M. (2009). Gyri –precise head model of transcranial DC stimulation: improved spatial focality using a ring electrode versus conventional rectangular pad. *Brain Stimul.* 2 201–207. 10.1016/j.brs.2009.03.005 20648973 PMC2790295

[B12] Datta A. Bikson M. Fregni F. (2010). Transcranial direct current stimulation in patients with skull defects and skull plates: high-resolution computational FEM study of factors altering cortical current flow. *Neuroimage* 52 1268–1278. 10.1016/j.neuroimage.2010.04.252 20435146 PMC2910315

[B13] Datta A. Zhou X. Su Y. Parra L. C. Bikson M. (2013). Validation of finite element model of transcranial electrical stimulation using scalp potentials: implications for clinical dose. *J. Neural Eng.* 10:036018. 10.1088/1741-2560/10/3/036018 23649036

[B14] Dmochowski J. P. Datta A. Huang Y. Richardson J. D. Bikson M. Fridriksson J. (2013). Targeted transcranial direct current stimulation for rehabilitation after stroke. *Neuroimage* 75 12–19. 10.1016/j.neuroimage.2013.02.049 23473936 PMC4120279

[B15] Ducharme S. Albaugh M. D. Nguyen T.-V. Hudziak J. J. Mateos-Pérez J. M. Labbe A. (2016). Trajectories of cortical thickness maturation in normal brain development–The importance of quality control procedures. *Neuroimage* 125 267–279. 10.1016/j.neuroimage.2015.10.010 26463175 PMC4691414

[B16] Fiocchi S. Ravazzani P. Priori A. Parazzini M. (2016). Cerebellar and spinal direct current stimulation in children: computational modeling of the induced electric field. *Front. Hum. Neurosci.* 10:522. 10.3389/fnhum.2016.00522 27799905 PMC5065976

[B17] Fischl B. van der Kouwe A. Destrieux C. Halgren E. Ségonne F. Salat D. H. (2004). Automatically parcellating the human cerebral cortex. *Cereb. Cortex* 14 11–22. 10.1093/cercor/bhg087 14654453

[B18] Geuzaine C. Remacle J.-F. (2009). Gmsh: a three-dimensional finite element mesh generator with built-in pre- and post-processing facilities. *Int. J. Numer. Methods Eng.* 79 1309–1331. 10.1002/nme.2579

[B19] Giedd J. N. Blumenthal J. Jeffries N. O. Castellanos F. X. Liu H. Zijdenbos A. (1999). Brain development during childhood and adolescence: a longitudinal MRI study. *Nat. Neurosci.* 2 861–863. 10.1038/13158 10491603

[B20] Gillick B. T. Kirton A. Carmel J. B. Minhas P. Bikson M. (2014). Pediatric stroke and transcranial direct current stimulation: methods for rational individualized dose optimization. *Front. Hum. Neurosci.* 8:739. 10.3389/fnhum.2014.00739 25285077 PMC4168687

[B21] Giorgio A. Watkins K. E. Chadwick M. James S. Winmill L. Douaud G. (2010). Longitudinal changes in grey and white matter during adolescence. *Neuroimage* 49 94–103. 10.1016/j.neuroimage.2009.08.003 19679191

[B22] Groeschel S. Vollmer B. King M. D. Connelly A. (2010). Developmental changes in cerebral grey and white matter volume from infancy to adulthood. *Int. J. Dev. Neurosci.* 28 481–489. 10.1016/j.ijdevneu.2010.06.004 20600789

[B23] Hameed M. Q. Dhamne S. C. Gersner R. Kaye H. L. Oberman L. M. Pascual-Leone A. (2017). Transcranial magnetic and direct current stimulation in children. *Curr. Neurol. Neurosci. Rep.* 17:11. 10.1007/s11910-017-0719-0 28229395 PMC5962296

[B24] Hofer S. Frahm J. (2006). Topography of the human corpus callosum revisited–comprehensive fiber tractography using diffusion tensor magnetic resonance imaging. *Neuroimage* 32 989–994. 10.1016/j.neuroimage.2006.05.044 16854598

[B25] Huang Y. Liu A. A. Lafon B. Friedman D. Dayan M. Wang X. (2017). Measurements and models of electric fields in the in vivo human brain during transcranial electric stimulation. *eLife* 6:e18834. 10.7554/eLife.18834 28169833 PMC5370189

[B26] Hummel F. Celnik P. Giraux P. Floel A. Wu W. H. Gerloff C. (2005). Effects of non-invasive cortical stimulation on skilled motor function in chronic stroke. *Brain* 128 490–499. 10.1093/brain/awh369 15634731

[B27] Huttenlocher P. R. (1979). Synaptic density in human frontal cortex - developmental changes and effects of aging. *Brain Res.* 163 195–205. 10.1016/0006-8993(79)90349-4 427544

[B28] Huttenlocher P. R. de Courten C. Garey L. J. Van der Loos H. (1982). Synaptogenesis in human visual cortex–evidence for synapse elimination during normal development. *Neurosci. Lett.* 33 247–252. 10.1016/0304-3940(82)90379-2 7162689

[B29] Jenkinson M. Beckmann C. F. Behrens T. E. J. Woolrich M. W. Smith S. M. (2012). FSL. *Neuroimage* 62 782–790. 10.1016/j.neuroimage.2011.09.015 21979382

[B30] Kessler S. K. Minhas P. Woods A. J. Rosen A. Gorman C. Bikson M. (2013). Dosage considerations for transcranial direct current stimulation in children: a computational modeling study. *PLoS One* 8:e76112. 10.1371/journal.pone.0076112 24086698 PMC3785412

[B31] Kirton A. (2017). Advancing non-invasive neuromodulation clinical trials in children: lessons from perinatal stroke. *Eur. J. Paediatr. Neurol.* 21 75–103. 10.1016/j.ejpn.2016.07.002 27470654

[B32] Kirton A. Ciechanski P. Zewdie E. Andersen J. Nettel-Aguirre A. Carlson H. (2016). Transcranial direct current stimulation for children with perinatal stroke and hemiparesis. *Neurology* 88 259–267. 10.1212/WNL.0000000000003518 27927938

[B33] Kirton A. Ciechanski P. Zewdie E. Andersen J. Nettel-Aguirre A. Carlson H. (2017). Transcranial direct current stimulation for children with perinatal stroke and hemiparesis. *Neurology* 88 259–267. 10.1212/WNL.0000000000003518 27927938

[B34] Klein A. Andersson J. Ardekani B. A. Ashburner J. Avants B. Chiang M.-C. (2009). Evaluation of 14 nonlinear deformation algorithms applied to human brain MRI registration. *Neuroimage* 46 786–802. 10.1016/j.neuroimage.2008.12.037 19195496 PMC2747506

[B35] Laakso I. Tanaka S. Mikkonen M. Koyama S. Sadato N. Hirata A. (2016). Electric fields of motor and frontal tDCS in a standard brain space: a computer simulation study. *Neuroimage* 137 140–151. 10.1016/j.neuroimage.2016.05.032 27188218

[B36] Lebel C. Walker L. Leemans A. Phillips L. Beaulieu C. (2008). Microstructural maturation of the human brain from childhood to adulthood. *Neuroimage* 40 1044–1055. 10.1016/j.neuroimage.2007.12.053 18295509

[B37] Lefaucheur J.-P. (2016). A comprehensive database of published tDCS clinical trials (2005-2016). *Neurophysiol. Clin.* 46 319–398. 10.1016/j.neucli.2016.10.002 27865707

[B38] Li Z. Park B.-K. Liu W. Zhang J. Reed M. P. Rupp J. D. (2015). A statistical skull geometry model for children 0-3 years old. *PLoS One* 10:e0127322. 10.1371/journal.pone.0127322 25992998 PMC4436309

[B39] Liebetanz D. Koch R. Mayenfels S. König F. Paulus W. Nitsche M. A. (2009). Safety limits of cathodal transcranial direct current stimulation in rats. *Clin. Neurophysiol.* 120 1161–1167. 10.1016/j.clinph.2009.01.022 19403329

[B40] Lindenberg R. Renga V. Zhu L. L. Nair D. Schlaug G. (2010). Bihemispheric brain stimulation facilitates motor recovery in chronic stroke patients. *Neurology* 75 2176–2184. 10.1212/WNL.0b013e318202013a 21068427 PMC3013585

[B41] Minhas P. Bikson M. Woods A. J. Rosen A. R. Kessler S. K. (2012). “Transcranial direct current stimulation in pediatric brain: a computational modeling study,” in *Proceedings of the Engineering in Medicine and Biology Society (EMBC), 2012 Annual International Conference of the IEEE*, San Diego, CA, 859–862. 10.1109/EMBC.2012.6346067 PMC3641645 23366028

[B42] Moliadze V. Lyzhko E. Schmanke T. Andreas S. Freitag C. M. Siniatchkin M. (2018). 1 mA cathodal tDCS shows excitatory effects in children and adolescents: insights from TMS evoked N100 potential. *Brain Res. Bull.* 140 43–51. 10.1016/j.brainresbull.2018.03.018 29625151

[B43] Moliadze V. Schmanke T. Andreas S. Lyzhko E. Freitag C. M. Siniatchkin M. (2015). Stimulation intensities of transcranial direct current stimulation have to be adjusted in children and adolescents. *Clin. Neurophysiol.* 126 1392–1399. 10.1016/j.clinph.2014.10.142 25468234

[B44] Opitz A. Paulus W. Will S. Antunes A. Thielscher A. (2015). Determinants of the electric field during transcranial direct current stimulation. *Neuroimage* 109 140–150. 10.1016/j.neuroimage.2015.01.033 25613437

[B45] Opitz A. Windhoff M. Heidemann R. M. Turner R. Thielscher A. (2011). How the brain tissue shapes the electric field induced by transcranial magnetic stimulation. *Neuroimage* 58 849–859. 10.1016/j.neuroimage.2011.06.069 21749927

[B46] Parazzini M. Fiocchi S. Liorni I. Priori A. Ravazzani P. (2014). Computational modeling of transcranial direct current stimulation in the child brain: implications for the treatment of refractory childhood focal epilepsy. *Int. J. Neural Syst.* 24:1430006. 10.1142/S012906571430006X 24475898

[B47] Parazzini M. Fiocchi S. Liorni I. Ravazzani P. (2015). Effect of the interindividual variability on computational modeling of transcranial direct current stimulation. *Comput. Intell. Neurosci.* 2015:963293. 10.1155/2015/963293 26265912 PMC4523656

[B48] Paus T. (2005). Mapping brain maturation and cognitive development during adolescence. *Trends Cogn. Sci.* 9 60–68. 10.1016/j.tics.2004.12.008 15668098

[B49] Reis J. Fritsch B. (2011). Modulation of motor performance and motor learning by transcranial direct current stimulation. *Curr. Opin. Neurol.* 24 590–596. 10.1097/WCO.0b013e32834c3db0 21968548

[B50] Reis J. Schambra H. M. Cohen L. G. Buch E. R. Fritsch B. Zarahn E. (2009). Noninvasive cortical stimulation enhances motor skill acquisition over multiple days through an effect on consolidation. *Proc. Natl. Acad. Sci. U.S.A.* 106 1590–1595. 10.1073/pnas.0805413106 19164589 PMC2635787

[B51] Shaw P. Kabani N. J. Lerch J. P. Eckstrand K. Lenroot R. Gogtay N. (2008). Neurodevelopmental trajectories of the human cerebral cortex. *J. Neurosci.* 28 3586–3594. 10.1523/JNEUROSCI.5309-07.2008 18385317 PMC6671079

[B52] Sowell E. R. Thompson P. M. Leonard C. M. Welcome S. E. Kan E. Toga A. W. (2004). Longitudinal mapping of cortical thickness and brain growth in normal children. *J. Neurosci.* 24 8223–8231. 10.1523/JNEUROSCI.1798-04.2004 15385605 PMC6729679

[B53] Thielscher A. Antunes A. Saturnino G. B. (2015). “Field modeling for transcranial magnetic stimulation: a useful tool to understand the physiological effects of TMS?,” in *Proceedings of the 2015 37th Annual International Conference of the IEEE Engineering in Medicine and Biology Society (EMBC)*, Milan, 222–225. 10.1109/EMBC.2015.7318340 26736240

[B54] Vines B. W. Cerruti C. Schlaug G. (2008). Dual-hemisphere tDCS facilitates greater improvements for healthy subjects’ non-dominant hand compared to uni-hemisphere stimulation. *BMC Neurosci.* 9:103. 10.1186/1471-2202-9-103 18957075 PMC2584652

[B55] Wendel K. Malmivuo J. (2006). Correlation between live and post mortem skull conductivity measurements. *Conf. Proc. IEEE Eng. Med. Biol. Sci.* 1 4285–4288. 10.1109/IEMBS.2006.259434 17947075

[B56] Yousry T. A. Schmid U. D. Alkadhi H. Schmidt D. Peraud A. Buettner A. (1997). Localization of the motor hand area to a knob on the precentral gyrus. A new landmark. *Brain J. Neurol.* 120(Pt 1), 141–157. 10.1093/brain/120.1.141 9055804

