# Supplementary material for: Modeling Transcranial Direct-Current Stimulation-Induced Electric Fields in Children and Adults
Source: Front Hum Neurosci. 2018 Jul 3;12:268. doi: 10.3389/fnhum.2018.00268 (PMC6037769; doi:10.3389/fnhum.2018.00268)
Supplement: Supplementary file 1 [file Data_Sheet_1.DOCX]

**Supplementary figures**

**
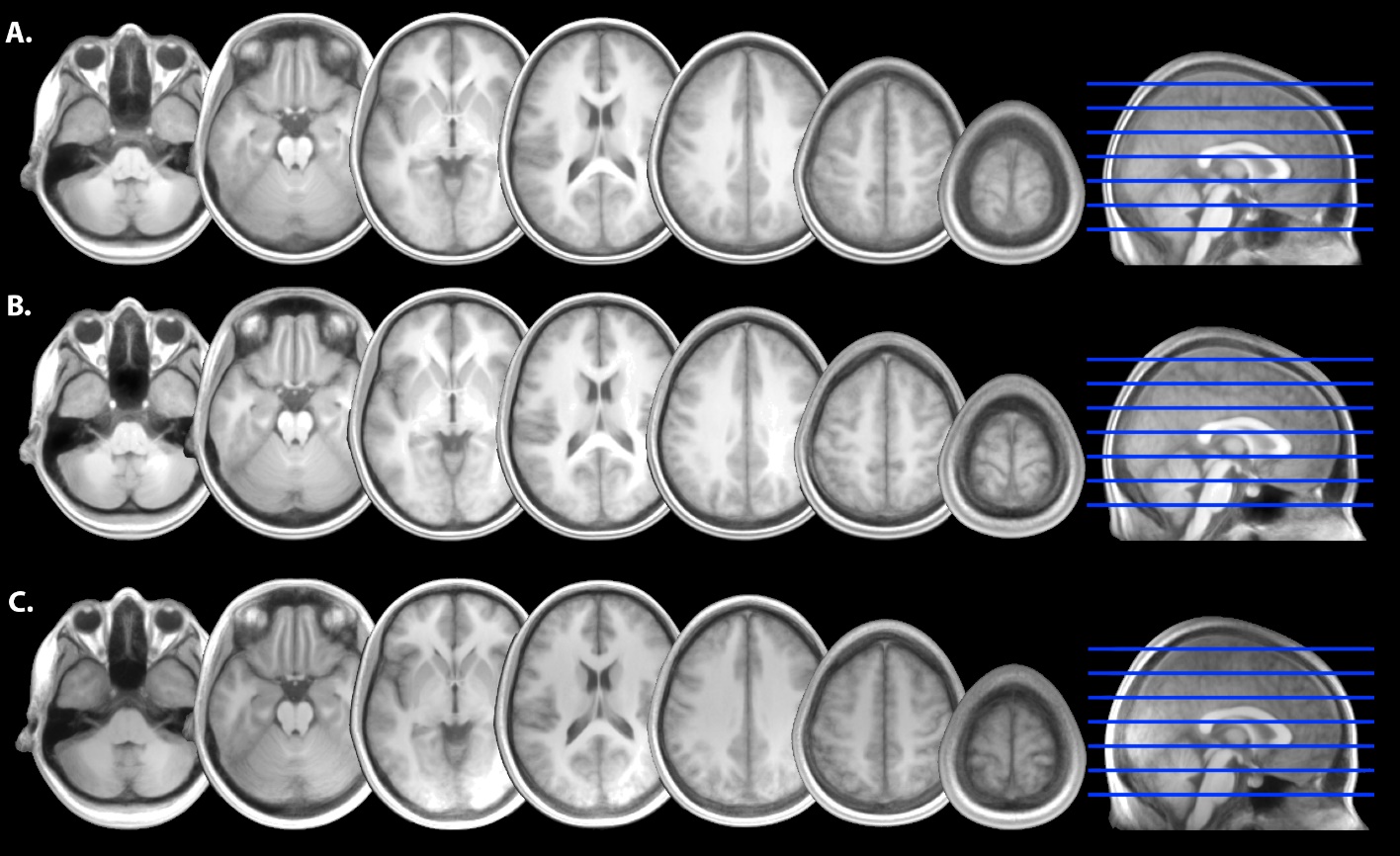
Figure S1**. A selection of axial slices from normalized group mean T1-weighted anatomical images for (A.) Child, (B.) Adolescents, and (C.) Adult sample groups. Each participants’ normalized T1-weighted anatomical image was overlaid on the MNI152 template and visually inspected to ensure accurate normalization.


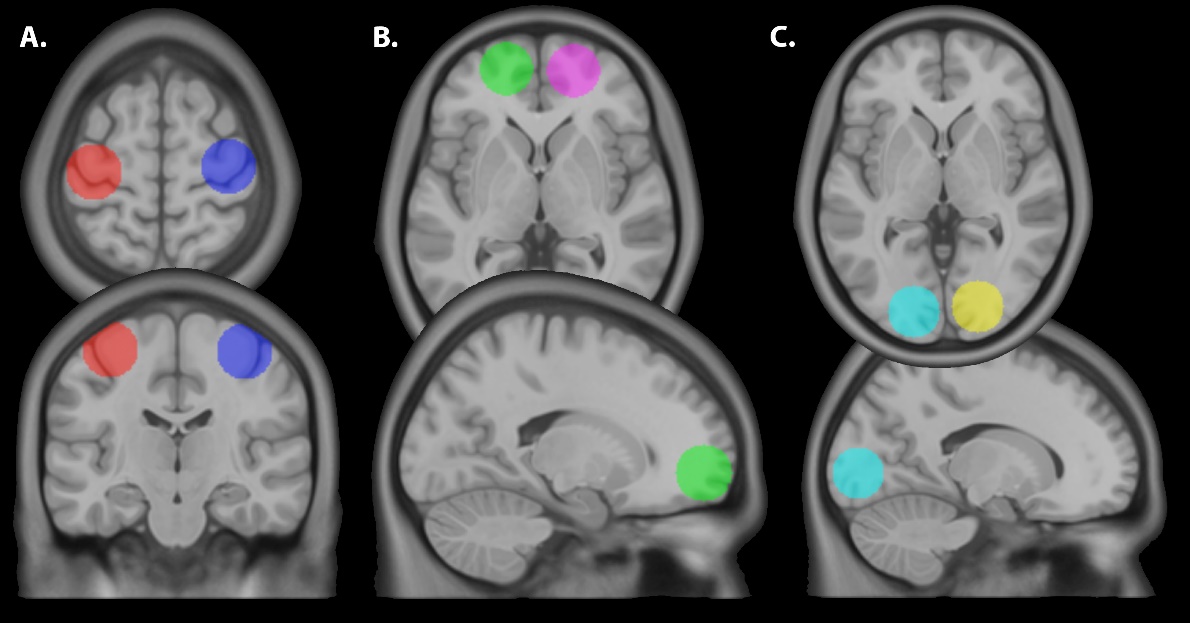


**Figure S2**. Spherical regions of interest were placed in (A.) bilateral primary motor, (B.) bilateral ventromedial prefrontal and (C.) primary visual cortices. Spheres are shown overlaid on the MNI152 template, were constrained to white and grey matter (i.e., do not include skull or CSF) and were 30 mm in diameter.


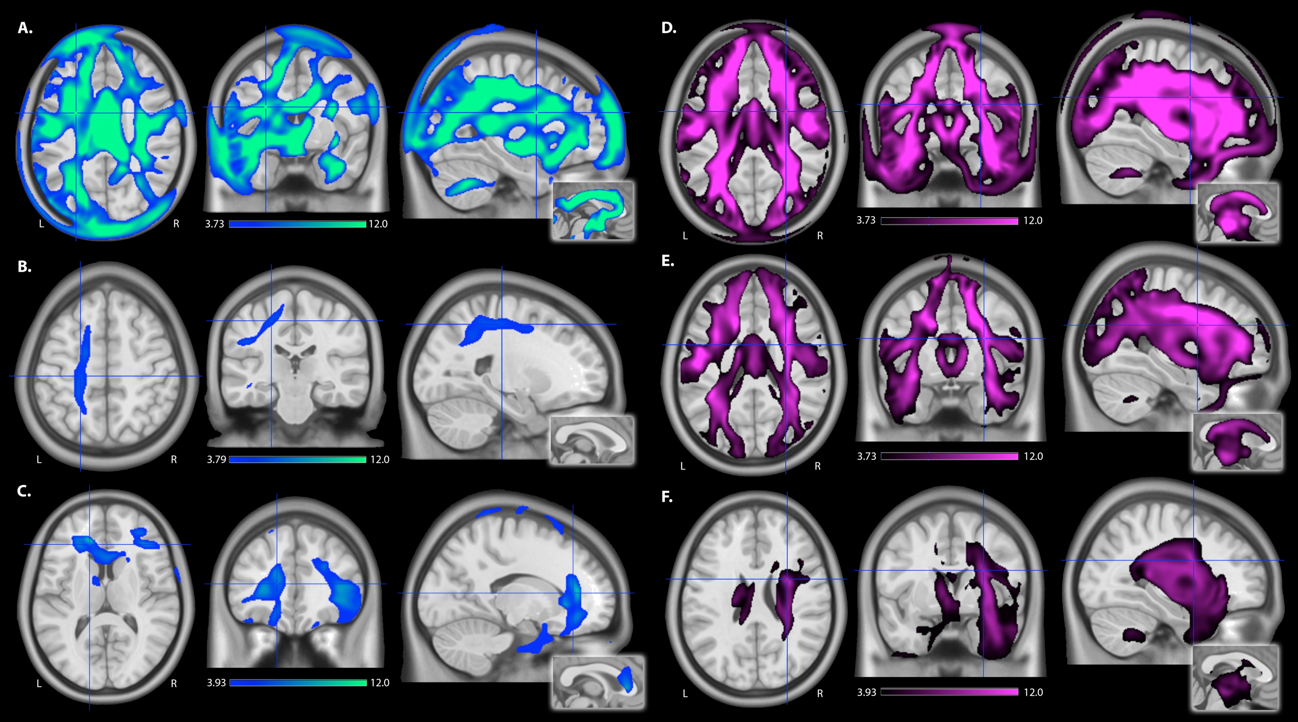


**Figure S3.** Areas of significantly higher estimated electric field (EF) strength when using individualized, anisotropic WM tensors in the current model compared to using a single isotropic conductivity value for all participants (paired t-test). Shown are statistical T-score heat maps for the cathodal (left panels A-C) and bihemispheric (right panels D-F) montages in three age groups (A&D.) Children, (B&E.) Adolescents, (C&F.) Adults. Insets for sagittal slices illustrate estimated EF strength differences in the corpus callosum at midline. T-score significance thresholds change slightly in each panel due to differences in group sample sizes.
